# Pressurized CO_2_ as a carboxylating agent for the biocatalytic *ortho*-carboxylation of resorcinol†
†Electronic supplementary information (ESI) available. See DOI: 10.1039/c8gc00008e


**DOI:** 10.1039/c8gc00008e

**Published:** 2018-04-03

**Authors:** Katharina Plasch, Gerhard Hofer, Walter Keller, Sam Hay, Derren J. Heyes, Alexander Dennig, Silvia M. Glueck, Kurt Faber

**Affiliations:** a Department of Chemistry , Organic & Bioorganic Chemistry , University of Graz , Heinrichstrasse 28 , 8010 Graz , Austria . Email: Si.Glueck@Uni-Graz.at ; Email: Kurt.Faber@Uni-Graz.at; b Institute of Molecular Biosciences , University of Graz , Humboldstrasse 50 , 8010 Graz , Austria; c Manchester Institute of Biotechnology , University of Manchester , 131 Princess Street , Manchester M1 7DN , UK; d Institute of Biotechnology and Biochemical Engineering , Graz University of Technology , Petersgasse 12 , 8010 Graz , Austria; e Austrian Centre of Industrial Biotechnology (ACIB) , Petersgasse 14 , 8010 Graz , Austria

## Abstract

The utilization of gaseous carbon dioxide instead of bicarbonate would greatly facilitate process development for enzyme catalyzed carboxylations on a large scale. As a proof-of-concept, 1,3-dihydroxybenzene (resorcinol) was carboxylated in the *ortho*-position using pressurized CO_2_ (∼30–40 bar) catalyzed by *ortho*-benzoic acid decarboxylases with up to 68% conversion. Optimization studies revealed tight pH-control and enzyme stability as the most important determinants.

## Abstract 2

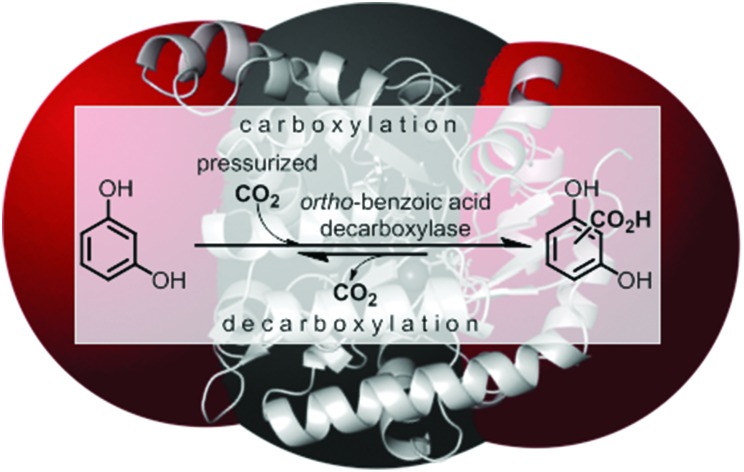
Utilization of gaseous carbon dioxide as a C_1_-building block in the biocatalytic *ortho*-carboxylation of a phenol.

## Introduction

Great efforts are currently undertaken to utilize the inexpensive, non-toxic and abundantly available waste gas CO_2_ as a C_1_ carbon source for the syntheses of valuable chemicals, materials or fuels.^1^ However, despite the fact that photosynthetic CO_2_-fixation mediated by RuBisCO is one of the most dominant reactions in nature, which binds ∼10^11^ tons CO_2_ p.a.,^2^ the chemical activation of CO_2_ remains challenging due to the high energy input required for substrate activation.^1^ Recently, the catalytic carboxylation of epoxides using salen complexes, zinc salts and double metal cyanide catalysts opened access to poly(ether)-carbonates for the production of polyurethanes^3^ and is at the threshold of industrial implementation. Further carboxylation strategies using (transition) metal-4 or organocatalysts^5^ have been developed to broaden the usability of carbon dioxide, but these methods are still in their infancy regarding commercialization.

The harsh reaction conditions (∼90 bar, 120–300 °C), varying *ortho*/*para*-selectivity and incomplete yields are major issues in the large-scale production of salicylic acids *via* the chemical carboxylation of phenolates using pressurized CO_2_ gas (Kolbe–Schmitt reaction).^6^ Although improved by microwave-heating using a bicarbonate-based ionic liquid, the process still suffers from moderate selectivity and yields.^7^

Biocatalytic methods have been explored as alternatives for the carboxylation of electron-rich (hetero)aromatic compounds to yield the corresponding carboxylic acids.^8^ Mild reaction conditions, exquisite regioselectivity and excellent yields (*e.g.* 95% for the bio-carboxylation of resveratrol)^9^ emphasize the power of bio-carboxylation processes.

**Fig. 1:**
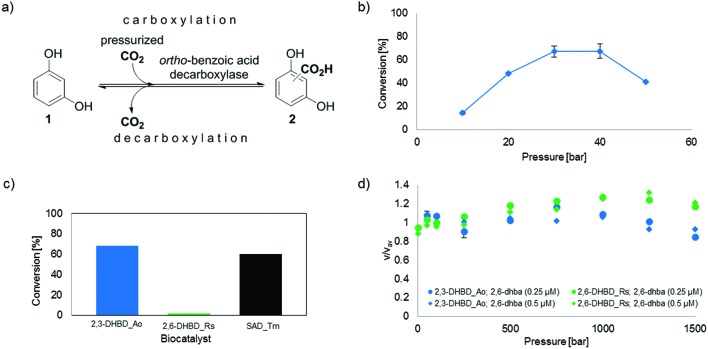
(a) Enzyme-catalyzed de/carboxylation of resorcinol (**1**). Carboxylated product **2** is a mixture of regio-isomeric 2,6- (2,6-dhba, **2a**) and 2,4-dihydroxybenzoic acid (2,4-dhba, **2b**) with a ratio of 3 : 4;10*b* (b) CO_2_ pressure dependence of the carboxylation of **1** using 2,3-DHBD_*Ao*; (c) carboxylation activity of decarboxylases with CO_2_ (30 bar) using **1** as a substrate; (d) stopped-flow measurements of the decarboxylation of 2,6-dhba (**2a**) with pressure-pretreated (≤1.5 kbar) 2,3-DHBD_*Ao* and 2,6-DHBD_*Rs*.

However, in the majority of biocatalytic carboxylation protocols reported so far, bicarbonate is used as a CO_2_ source, which needs to be applied at elevated concentrations (∼3 M) to shift the equilibrium towards the thermodynamically unfavored carboxylation.^10^ In practice, excess bicarbonate is not only wasteful, but also creates problems during work-up (foaming) upon acidification. In contrast, the use of alternative CO_2_ sources, such as pressurized or sub/supercritical CO_2_ for biocatalytic carboxylation is not well investigated. So far, biocatalytic carboxylations were only successful when additional HCO_3_^–^ (2–3 M) was applied.^11^ In order to develop an operationally simple protocol amenable to scale-up, the use of pressurized CO_2_ gas was investigated in the carboxylation of 1,3-dihydroxybenzene (**1**, resorcinol, Fig. 1a) as a test substrate using 2,3-dihydroxybenzoic acid decarboxylase from *Aspergillus oryzae* (2,3-DHBD_*Ao*),^12^ 2,6-dihydroxybenzoic acid decarboxylase from *Rhizobium* sp. (2,6-DHBD_*Rs*)^13^ and salicylic acid decarboxylase from *Trichosporon moniliiforme* (SAD_*Tm*),10*a* which are highly active in the presence of bicarbonate.^9^–^11^ Special emphasis was devoted to pressure and pH effects on enzyme stability.

The exposure of enzymes to scCO_2_‡
‡scCO_2_ = supercritical carbon dioxide. pressure has an impact on activity, stability or selectivity,^14^ which is either due to conformational changes in their secondary and tertiary structure^15^ or due to the chemical modification of basic amino acid residues (*e.g.* Lys, Arg, His) by *N*-carboxylation forming carbamates.^16^ The most prominent is the carboxylation of lysine residues (*e.g.* in RuBisCO,^17^ urease^18^), which is required for structural reasons (*e.g.* ligand for binding of metal ions in RuBisCO)^17^ or even mandatory for catalysis (*e.g.* β-lactamase OXA-10 from *Pseudomonas aeruginosa*,^19^ biotin-dependent enzymes^20^). In contrast to these rare beneficial effects, the scCO_2_ treatment of enzymes was reported to cause a decrease or complete loss of enzyme activity due to enforced conformational changes (*e.g.* horseradish peroxidase,15*b* lipase,15*a* tyrosinase15*d*).

## Experimental

### General

Resorcinol (**1**) and 2,6-dihydroxybenzoic acid (**2a**) were purchased from Sigma Aldrich and 2,4-dihydroxybenzoic acid (**2b**) was obtained from Fluka Analytical. SYPRO orange was purchased from Invitrogen. The pressure reactor system (DigiCAT-system from the HEL Group, volume 16 mL) was equipped with a gas inlet and a magnetic stirrer. A HisTrapFF column with a Ni-NTA Matrix and a PD10 desalting column were obtained from GE Healthcare and Vivaspin 20 (30 kDa) was obtained from Sartorius AG. CO_2_ gas (3.0 = 99.9% purity) was obtained from the Linde Group. High pressure stopped-flow measurements were performed with a Hi-Tech Scientific HPSF-56 high pressure stopped-flow spectrophotometer from TgK Scientific. A thermal cycler and a CFX real time system for fluorescence measurements were from Bio Rad and a WebQC calculator was used for pH-calculations.^21^ 2,3-Dihydroxybenzoic acid decarboxylase from *Aspergillus oryzae* (2,3-DHBD_*Ao*), 2,6-dihydroxybenzoic acid decarboxylase from *Rhizobium* sp. (2,6-DHBD_*Rs*) and salicylic acid decarboxylase from *Trichosporon moniliiforme* (SAD_*Tm*) were cloned and overexpressed as previously described.^22^

### General procedure for biotransformation under pressurized CO_2_ gas

Lyophilized whole cells (90 mg *E. coli* host cells containing the corresponding overexpressed enzyme with an activity of 5.7 ± 0.7 U mg^–1^ 2,3-DHBD_*Ao* and 38.1 ± 0.8 U mg^–1^ 2,6-DHBD_*Rs*, respectively) were rehydrated in TRIS-HCl buffer (2850 μL, pH 9.0, 100 mM) for 30 min. The substrate **1** [10 mM final concentration, dissolved in 150 μL MeOH (5% v/v)] was added to the enzyme solution (3 mL final volume) which was transferred into a pressure reactor. After CO_2_ gas (30 bar) was applied *via* an additional gas inlet for ∼1 h, the reaction mixture was stirred at 50 rpm for 24 h at 30 °C in the tightly sealed pressure reactor. After 24 h the reaction was stopped by taking 100 μL of the reaction mixture and diluting it in 900 μL of H_2_O/MeCN/trifluoroacetic acid (TFA, 50 : 50 : 3) to precipitate the enzyme, which was removed by centrifugation (10 min, 14 000 rpm). The supernatant was directly used for measurements on a reversed-phase HPLC system.

For CO_2_ pressure studies 10, 20, 30, 40 and 50 bar CO_2_ gas was applied.

For buffer concentration studies 100, 250 and 500 mM TRIS-HCl buffer was applied.

The CO_2_ pressure pretreatment experiments with 2,6-DHBD_*Rs* were performed under the same conditions as described above at 10, 40 and 50 bar CO_2_ gas (30 mg whole cells, 950 μL TRIS-HCl buffer, pH 9.0, 100 mM), however, without the addition of the substrate. The pressure pretreated enzyme was then used for the decarboxylation of 2,6-dhba **2a** [final concentration 10 mM, dissolved in 50 μL MeOH (5% v/v)] in a glass vial (1 mL final volume). The vials were tightly sealed with screw caps and samples were shaken for 24 h, 120 rpm at 30 °C.

For the determination of the enzymatic activity, the lyophilized whole cells of 2,3-DHBD_*Ao* and 2,6-DHBD_*Rs* (10 mg mL^–1^) were rehydrated in TRIS-HCl buffer (950 μL, pH 9.0, 100 mM) for 30 min. The substrate **2a** (10 mM final concentration) was added to the enzyme solution (1 mL final volume). The vials were shaken at 30 °C with 120 rpm for 0, 1, 2, 4, 6, 8, 10, 12, 15 and 20 min.

All screening experiments were carried out at least in triplicates and all reactor experiments at least in duplicates.

### High pressure stopped-flow system

High pressure stopped-flow measurements were performed under a pressure of 1 bar–1.5 kbar using a purified enzyme (0.5 μM 2,3-DHBD_*Ao* and 2,6-DHBD_*Rs*, respectively) in TRIS-HCl buffer (pH 9.0, 100 mM) with two different 2,6-dhba concentrations (0.25 and 0.5 μM **2a**, dissolved in 5% v/v MeOH) over 1 min at 30 °C. Spectral changes of the reaction were monitored at 320 nm. All screening experiments were carried out at least in triplicates.

### Thermostability experiments

For differential scanning fluorimetry, protein solution [10 μL, 0.2 g L^–1^ in 5 mM MES (pH 6), 150 mM NaCl], SYPRO orange (10 μL, 1 : 500 diluted in sterile ultrapure water) and multicomponent buffer (pH 4 to 9) (10 μL, 1 : 2 : 2 molar ratio of l-malic acid, MES and TRIS; 1 M total concentration)^23^ were mixed in 96 well plates. Using a C1000 thermal cycler, the solution was heated at 1.2 °C per minute, from 25 °C to 95 °C. Fluorescence was measured every 0.3 °C, using channel 2 of a CFX real time system. For the smaller step size experiment between pH 4 and 5, sodium citrate buffer (100 mM) was used and the temperature range extended from 10 °C to 95 °C. The melting temperature *T*_m_ was calculated as the minimum of the first derivative of the fluorescence *vs.* the temperature. All experiments were carried out in triplicates.

### Analytics

#### HPLC analysis

HPLC/UV experiments were performed on a HPLC Agilent 1260 Infinity system with a diode array detector and a reversed phase Phenomenex Luna column C18 (100 Å, 250 × 4.6 mm, 5 μm, column temperature 24 °C). Conversions were determined by comparison with calibration curves for products and substrates prepared with an authentic reference material. All compounds were spectrophotometrically detected at 263 nm. The method was run over 22 min with H_2_O/TFA (0.1%) as the mobile phase (flow rate 1 mL min^–1^) and a MeCN/TFA (0.1%) gradient (0–2 min 5%, 2–15 min 5–100%, 15–17 min 100%, 17–22 min 100–5%).

#### HR-MS analysis

HR-MS analysis was performed on a nanoHPLC (Ultimate 3000 RSLCnano system – Dionex) system coupled with q-TOF Maxis II-ETD with an ESI-ionisation in positive mode.

## Results and discussion

In spite of the previous reports that carboxylation of phenols requires significant concentrations of bicarbonate (∼0.5 M minimum, levelling off at ∼1 M to reach a flat plateau at ∼3 M) to achieve appreciable conversion levels,^11^ but fails with CO_2_ (gas) alone,11*b* we tested the use of commercial sparkling water as a medium for the carboxylation of resorcinol (**1**) using 2,3-DHBD_*Ao*. Surprisingly, conversions of 23% and 7% were measured in water samples containing only 55 mM or 4 mM HCO_3_^–^, respectively, which favorably compares to 22% obtained under standard conditions (3 M bicarbonate) (for details see the ESI†).

In order to evaluate the usability of CO_2_ (gas) for carboxylation, the influence of various levels of CO_2_ pressure (10–50 bar) on the conversion of resorcinol (**1**, 10 mM, TRIS-HCl buffer 100 mM, pH 9.0) using 2,3-DHBD_*Ao*^12^ was determined (Fig. 1b). A bell-shaped curve of the CO_2_ pressure with an optimum between 30 and 40 bar was found corresponding to a maximum conversion of 68% of carboxylated product (**2**). The conversion was very low below ≤10 bar and dropped significantly at 50 bar. A time study proved that under these conditions equilibrium was reached at ∼24 h (see ESI, Fig. S3†).

In order to examine whether pressurized CO_2_ gas (30 bar) is also accepted by other decarboxylases, 2,6-DHBD_*Rs*^13^ and SAD_*Tm*10*a* were tested (Fig. 1c). While SAD_*Tm* yielded similar results obtained with 2,3-DHBD_*Ao* (66% and 60% conv., respectively), 2,6-DHBD_*Rs* did not lead to an appreciable amount of carboxylated product (**2**, conv. <2%). This result corroborates a previous observation, that 2,6-DHBD_*Rs* is inactive under 50–80 bar of CO_2_.11*b*

To answer the question whether pressure *per se* (a physical consequence) or pressurized carbon dioxide (a chemical effect) is responsible for the inactivation of 2,6-DHBD_*Rs*, high pressure stopped-flow experiments were performed. For reasons of simplicity, the activity of (hydrostatic) pressure-pretreated 2,6-DHBD_*Rs* was determined in the (energetically favored) decarboxylation direction with **2a** as a substrate (Fig. 1d). The fairly constant velocity (*v*/*v*_av_ = 0.8–1.3) of substrate consumption (monitored by a decrease of absorbance at 320 nm) of both enzymes pretreated with up to 1.5 kbar reveals their general pressure stability (Fig. 1d, see also ESI, Fig. S5–S7†). Consequently, the inactivation of 2,6-DHBD_*Rs* can be explicitly assigned to the action of pressurized CO_2_.

**Fig. 2:**
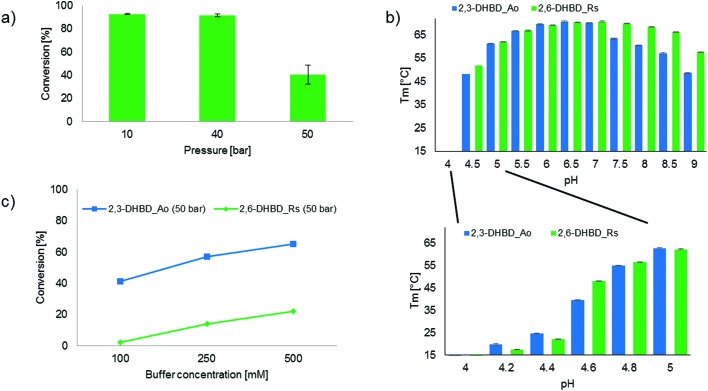
(a) Decarboxylation activity of CO_2_ pressure pretreated (10, 40 and 50 bar) 2,6-DHBD_*Rs* using **2a** as a substrate; (b) DSF comparison of the pH dependent melting temperature of 2,3-DHBD_*Ao* and 2,6-DHBD_*Rs* in multicomponent buffer (pH 4 to 9) and citrate buffer (pH 4 to 5); (c) Influence of TRIS-HCl buffer concentration on the carboxylation of **1** using 2,6-DHBD_*Rs* and 2,3-DHBD_*Ao* under 50 bar CO_2_ pressure.

In order to determine whether the CO_2_ dependent inactivation of 2,6-DHBD_*Rs* is reversible, the biocatalyst was pretreated with CO_2_ pressure (10, 40 and 50 bar, respectively) before measuring its decarboxylation activity (Fig. 2a). The sharp drop in conversion between pretreatments with 40 and 50 bar CO_2_ (92% *versus* 40% conv.) clearly indicates that 2,6-DHBD_*Rs* is irreversibly deactivated beyond ∼40 bar CO_2_.

Since carbamate formation *via* the carboxylation of lysine residues is a prime suspect for enzyme deactivation, HR-MS measurements were performed. However, no difference in mass between the native and CO_2_ pressure (50 bar) treated 2,6-DHBD_*Rs* was detected, thus inactivation is most likely not caused by the carboxylation of basic amino acid residues (see ESI, Fig. S4†).

Carbon dioxide is readily dissolved in the aqueous reaction medium leading to a drop in pH due to the dissociation of H_2_CO_3_.^24^ This effect was applied by Hofland *et al.*^25^ using CO_2_ gas as a ‘volatile acid’ within a range of pH 4–9 to precipitate proteins. To evaluate whether differences in pH-dependent structural stability between 2,3-DHBD_*Ao* and 2,6-DHBD_*Rs* could explain their disparate activity, differential scanning fluorimetry (DSF) experiments were performed with both proteins. A first experiment using a multi-component buffer system (l-malic acid, MES, TRIS)^23^ shows a broad pH-window from pH 4 to 9, while a second run using citrate buffer and smaller increments reveals details within the pH range of 4 to 5. Overall, 2,3-DHBD_*Ao* and 2,6-DHBD_*Rs* behave similarly over the whole pH range. Both are thermally most stable between pH 6–7 and show a continuous decrease in denaturation temperature upon higher or lower pH levels (Fig. 2b). Both enzymes are unstable already at room temperature when the pH of the medium reaches below 4.6.

Given that this pH is likely reached in water in a CO_2_ pressurized system (30 bar CO_2_ in 0.1 M TRIS-buffer corresponds to a calculated pH of 4.6),^21^ the influence of the buffer capacity was investigated. An increase of buffer concentration/capacity (TRIS-HCl buffer, 100, 250 and 500 mM) to compensate for acidification due to H_2_CO_3_ dissociation and product formation considerably improved the conversion of the carboxylation of **1** with both enzymes (2,3-DHBD_*Ao* ∼1.5-fold increase; 2,6-DHBD_*Rs* ∼10-fold increase) (Fig. 2c). These results as well as the DSF analysis clearly indicate that the pH value in the pressure chamber is at the edge of the operational pH-window for both enzymes, with 2,3-DHBD_*Ao* performing slightly better.

Since the economic usage of resources constitutes an important parameter, the atom economy of various *o*-carboxylation systems was compared (Table 1, see the ESI†). An excellent atom efficiency of 100% combined with a good yield (68%) verifies the benefit of the biocatalytic approach using CO_2_ (gas). By way of comparison, the biocatalytic alternative using high amounts of bicarbonate shows a significant drop in atom efficiency (73%), which further drops in the case of traditional chemical (52% and 61%, respectively) or microwave-assisted methods (55%).

**Table 1:** Comparison of the atom economy of various carboxylation methods of resorcinol (**1**)

Method	Yield [%]	Atom economy [%]	Ref.
Biocatalytic (CO_2_ gas)	68	100	This work
Biocatalytic (HCO_3_^–^)	22	73	22
Chemical (Kolbe–Schmitt)	56	52	6*b*
Chemical (Kolbe–Schmitt)	47	61	26
Chemical (microwave-assisted Kolbe–Schmitt)	62	55	7

## Conclusion

In summary, we have demonstrated that pressurized carbon dioxide can be used directly as a carboxylating agent in the enzyme catalyzed *o*-carboxylation of a phenol as an alternative to the high concentration of bicarbonate. Two enzyme candidates (2,3-DHBD_*Ao* and SAD_*Tm*) readily accepted the alternative CO_2_ source for the carboxylation of the model substrate resorcinol. In contrast, 2,6-DHBD_*Rs* was inapplicable under CO_2_ pressure due to irreversible inactivation, which was correlated to a decrease in pH caused by the dissociation of H_2_CO_3_.

Overall, the use of pressurized CO_2_ gas significantly improves the efficiency of biocatalytic carboxylations and facilitates downstream-processing of this benign and sustainable approach in using CO_2_ as a carbon feedstock for the synthesis of organic acids.

## Conflicts of interest

There are no conflicts of interest to declare.

## Supplementary Material

Supplementary information
