# Supplementary material for: Pressurized CO2 as a carboxylating agent for the biocatalytic ortho-carboxylation of resorcinol
Source: Green Chem. 2018 Apr 3;20(8):1754–9. doi: 10.1039/c8gc00008e (PMC5942041; doi:10.1039/c8gc00008e)
Supplement: Supplementary file 1 [file GC-020-C8GC00008E-s001.pdf]

Electronic Supplementary Material (ESI) for Green Chemistry.
This journal is © The Royal Society of Chemistry 2018

Pressurized CO_2 as Carboxylating Agent for the Biocatalytic *ortho*-Carboxylation of Resorcinol

Katharina Plasch,[b] Gerhard Hofer,[c] Walter Keller,[c] Sam Hay,[d] Derren Heyes,[d] Alexander Dennig,[e] Silvia M. Glueck,[a,b,*] Kurt Faber,[b,*]

[a] Austrian Centre of Industrial Biotechnology (ACIB), c/o

[b] Department of Chemistry, Organic & Bioorganic Chemistry, University of Graz, Heinrichstrasse 28, 8010 Graz, Austria

[c] Institute of Molecular Biosciences, University of Graz, Humboldstrasse 50, 8010 Graz, Austria

[d] Manchester Institute of Biotechnology, University of Manchester, 131 Princess Street, Manchester M1 7DN, United Kingdom

[e] Institute of Biotechnology and Biochemical Engineering, Technical University of Graz, Petersgasse 12, 8010 Graz, Austria

Electronic Supporting Information

1.	Purification of 2,6-DHBD_*Rs* and 2,3-DHBD_*Ao*	2
2.	Sparkling water study	2
2.1.	Sparkling water analysis	2
2.2.	Sparkling water screening	2
3.	Time study	3
4.	Evaluation of buffer system	4
5.	High-resolution MS	4
6.	High pressure stopped-flow measurements	5
6.1.	Kinetics	5
6.2.	High pressure stopped-flow results	6
7.	Atom economy calculations	6
7.1.	Biocatalytic carboxylation of resorcinol (**1**) with CO_2 pressure	6
8.	References	7

* Corresponding authors: <Si.Glueck@Uni-Graz.at>, <Kurt.Faber@Uni-Graz.at>

1. Purification of 2,6-dihydroxybenzoic acid decarboxylase from *Rhizobium* sp. (2,6-DHBD_*Rs*) and 2,3-dihydroxybenzoic acid decarboxylase from *Aspergillus oryzae* (2,3-DHBD_*Ao*)

Lyophilized *E. coli* cells containing the corresponding overexpressed enzyme (1 g) were rehydrated in lysis buffer (10 mM imidazole, 10 mL) for 30 min at 30 °C with 120 rpm. After ultrasonication (30% amplitude, 1 sec pulse, 4 sec pause, 5 min overall time) on ice, the cells were centrifuged (10 min at 4 °C with 18 000 rpm) and the supernatant was filtered (0.45 µm filter). For purification a HisTrapFF column with Ni-NTA matrix was used. After combining the fractions with the highest enzyme concentration (determined via Bradford assay) the fractions were centrifuged in a Vivaspin 20 (30 kDa) for 30 min at 4 °C with 4000 rpm. The purified enzymes were used for the bio-carboxylation after a buffer salt exchange with a PD10 desalting column in the TRIS-HCl buffer 50 mM, pH 7.5. The overexpression of the gene was analyzed via SDS-PAGE (Figure S1).

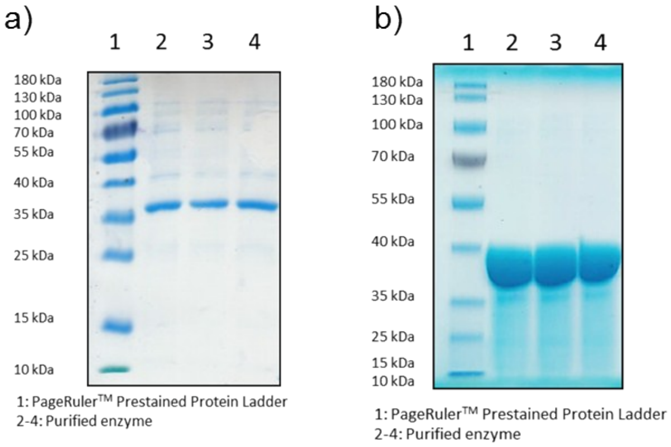

Figure S1. SDS-PAGE analysis of a) purified 2,6-DHBD_*Rs* and b) purified 2,3-DHBD_*Ao*.

2. Sparkling water study

2.1. *Sparkling water analysis*

Vöslauer®[1] (extract from full analysis) [mg L^{-1}]: K^+ 1.6, Na^+ 14.1, Mg^{2+} 40.7, Ca^{2+} 112.0, Cl^- 19.4, SO_4^{2-} 221.0, HCO_3^- 259.0, NO_3^- <1.0, *m*-silicic acid 14.1, F^- 0.7, dissolved solids 682.6.

Sicheldorfer®[2] (extract from full analysis) [mg L^{-1}]: K^+ 108.8, Na^+ 1175.0, Mg^{2+} 87.3, Ca^{2+} 175.1, HCO_3^- 3400.0, I^- 0.8.

2.2. *Sparkling water screening*

For biotransformations with sparkling water as reaction medium lyophilized whole cells (30 mg, *E. coli* host cells containing overexpressed 2,3-DHBD_*Ao*) were rehydrated in sparkling water (900 µL of Sicheldorfer®, Vöslauer®). The substrate **1** [10 mM final concentration, dissolved in 50 µL

MeOH (5% v/v)] was added to the enzyme solution (1 mL final volume) which was transferred into a glass vial. The vials were tightly sealed with screw caps and were shaken for 24 h at 120 rpm and 30 °C.

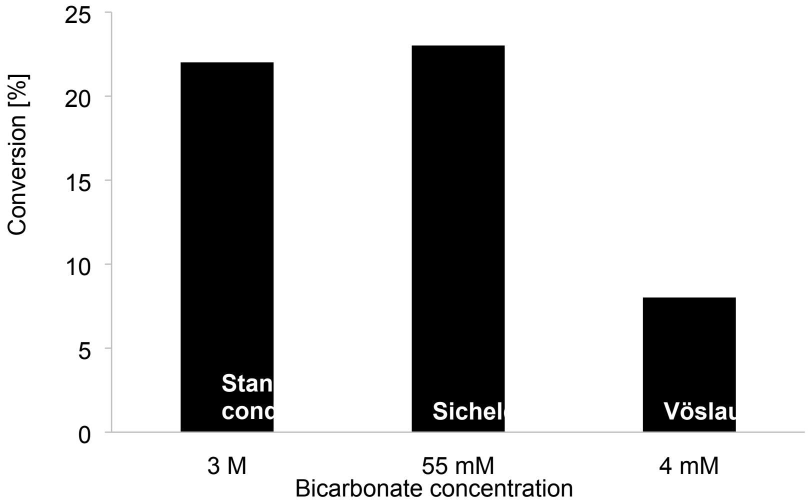

Figure S2. Conversion of the carboxylation of resorcinol (**1**) using 2,3-DHBD_*Ao* under standard conditions (3 M $KHCO_3$) and in sparkling water.

3. Time study

Lyophilized whole cells (90 mg) were rehydrated in TRIS-HCl buffer (2850 µL, pH 9.0, 100 mM) and substrate **1** (10 mM final concentration) was added to the enzyme solution (3 mL final volume). The pressure reactor was tightly sealed and the reaction mixture was stirred at 50 rpm and 30 °C for 0, 1, 4, 24, 30 and 48 h.

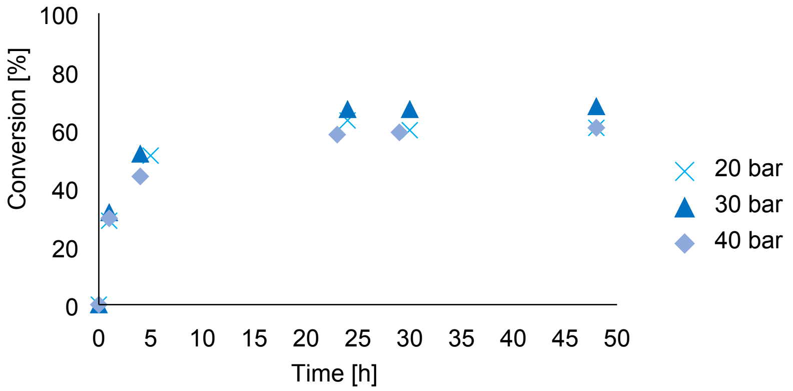

Figure S3. Evaluation of the reaction time for the carboxylation of resorcinol (**1**) with 2,3-DHBD_*Ao* under 20, 30 and 40 bar CO_2 pressure within 0 – 48 h.

4. Evaluation of buffer system

In order to achieve higher conversions, the buffer system was changed from a P_i buffer 100 mM, pH 5.5 to a TRIS-HCl buffer 100 mM, pH 9.0 (20 bar CO_2, 30 °C, 50 rpm, 24 h).

Table S1. pH study using TRIS-HCl buffer (100 mM, pH 9.0) and phosphate buffer (100 mM, pH 5.5) under 20 bar CO_2 with the model substrate resorcinol (**1**) and 2,3-DHBD_*Ao*.

buffer system	conversion [%]
Phosphate buffer, pH 5.5, 100 mM	19
TRIS-HCl buffer, pH 9.0, 100 mM	58

5. High-resolution MS

500 µL of purified enzyme (2,6-DHBD_*Rs*) was treated under CO_2 pressure (50 bar) for 24 h at 30 °C and 50 rpm in the pressure reactor in the absence of substrate. The final protein concentration was defined via Bradford assay and the sample was diluted with TRIS-HCl buffer (50 mM, pH 7.5) to a final concentration of 1 mg mL^{-1}. As reference the native purified enzyme was used.

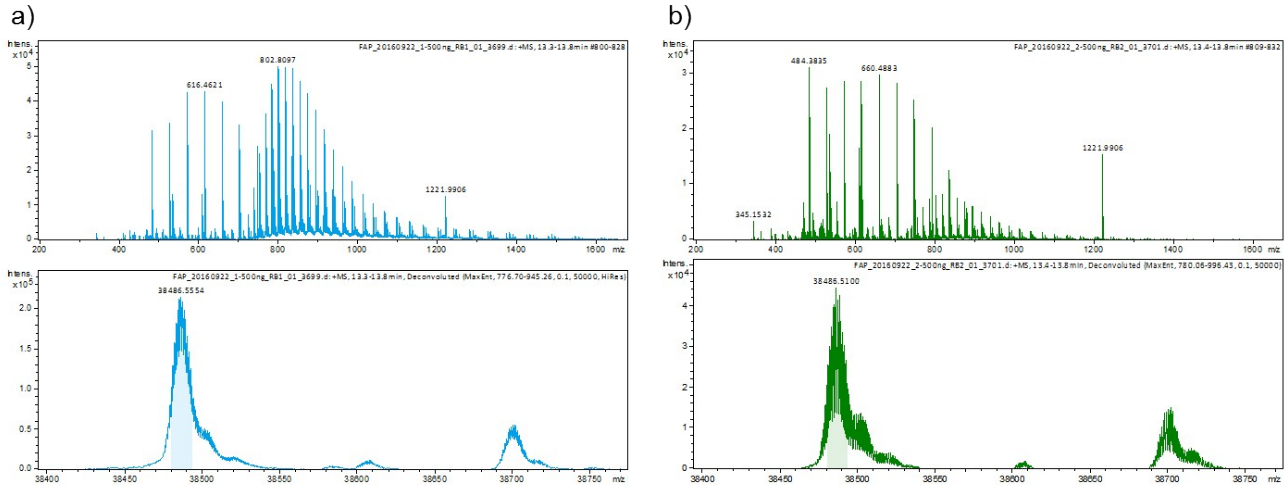

Figure S4. HR-MS chromatogram of a) native 2,6-DHBD_*Rs* (calc.: 37398,92, found: 38486,5554) and b) 50 bar CO_2 pressure-treated 2,6-DHBD_*Rs* (calc.: 37398,92, found: 38486,51).

6. High pressure stopped-flow system

6.1. *Kinetics*

Calculations of kinetic parameters for the high pressure stopped-flow measurements were done with 0.5 µM enzyme and 0 – 750 µM 2,6-dihydroxybenzoic acid (**2a**) as substrate (Figure S5 and Figure S6).

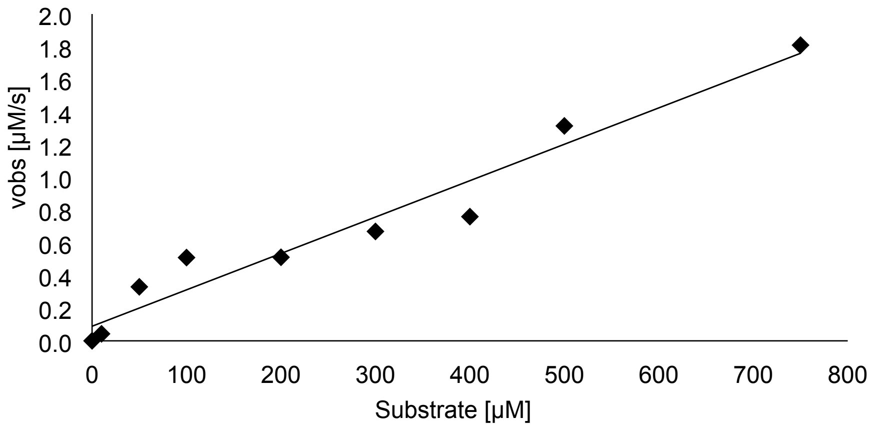

Figure S5. Plot of velocity vs. substrate concentration; 2,6-dhba (**2a**, 0 – 750 µM) with 0.5 µM 2,3-DHBD_*Ao* as catalyst; K_m = 0.4 mM, k_{cat} = 3.6 s^{-1}.

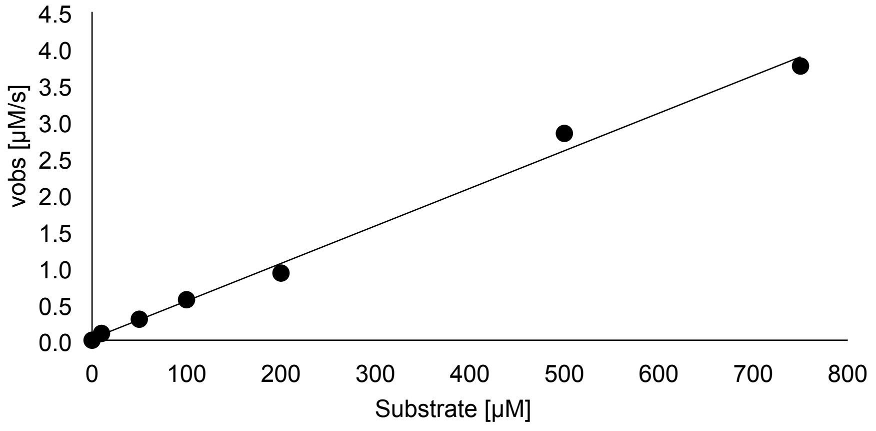

Figure S6. Plot of velocity vs. substrate concentration; 2,6-dhba (**2a**, 0 – 750 µM) with 0.5 µM 2,6-DHBD_*Rs* as catalyst; K_m = 0.4 mM, k_{cat} = 7.5 s^{-1}.

6.2. *Stopped flow high-pressure results*

Stopped flow high-pressure measurements with 2,3-DHBD_*Ao* and 2,6-DHBD_*Rs* were performed over 1 min at 30 °C at 1 bar – 1.5 kbar. Figure S7 shows the velocity (v_{obs} [µM s^{-1}]) of each experiment at different pressure.

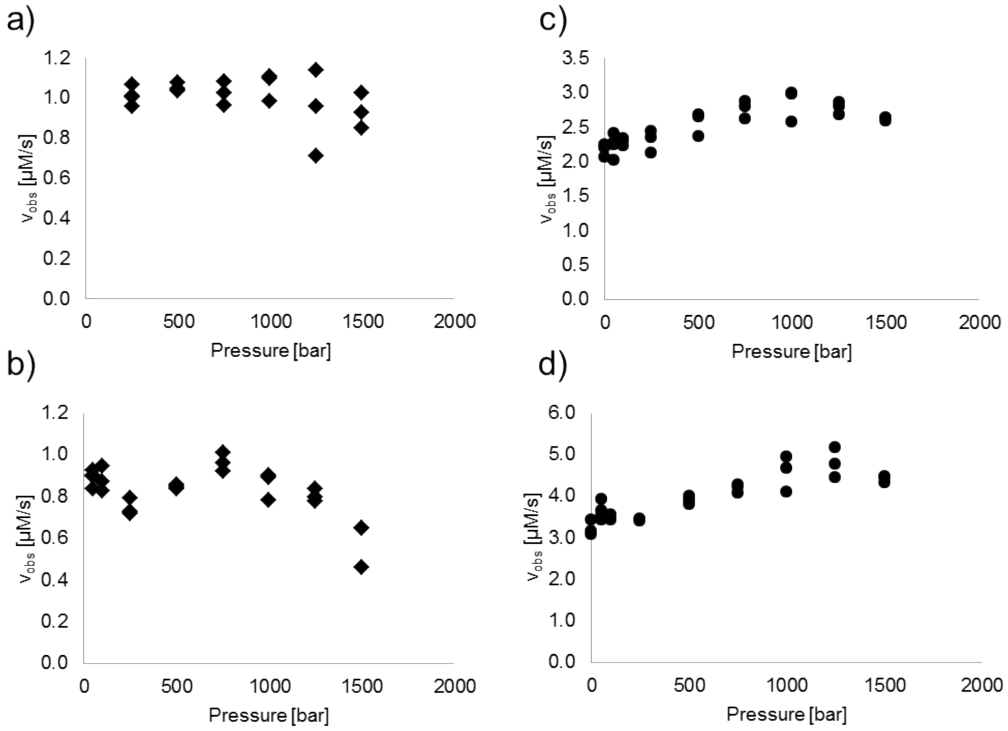

Figure S7. Stopped flow high-pressure measurements of 2,3-DHBD_*Ao* and 2,6-DHBD_*Rs* (0.5 µM) with 2,6-dhba (**2a**, 250 and 500 µM, resp.) at 320 nm (v_{obs} [µM s^{-1}]). a) 0.5 µM 2,3-DHBD_*Ao* and 500 µM 2,6-dhba (**2a**); b) 0.5 µM 2,3-DHBD_*Ao* and 250 µM 2,6-dhba (**2a**); c) 0.5 µM 2,6-DHBD_*Rs* and 500 µM 2,6-dhba (**2a**); d) 0.5 µM 2,6-DHBD_*Rs* and 250 µM 2,6-dhba (**2a**).

7. Atom economy calculations

$$atom\ economy = \frac{molecular\ mass\ of\ desired\ product}{molecular\ mass\ of\ all\ reactants} x100$$

7.1. Biocatalytic carboxylation of resorcinol (1) with CO_2 pressure

$$atom\ economy = \frac{154.12\frac{g}{mol}}{110.11\frac{g}{mol} + 44.01\frac{g}{mol}} x100$$

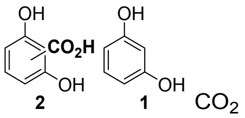

The atom economy of all carboxylation methods are summarized in Table 1 (main paper).

8. References

[1] http://www.voeslauer.com/web/at/quelle/quellanalyse (07.10.2016)

[2] http://www.heilwassersicheldorf.com/#block-block-9 (5.2.2017)
